# Radiofrequency for the Treatment of Lumbar Radicular Pain: Impact on Surgical Indications

**DOI:** 10.1155/2015/392856

**Published:** 2015-08-16

**Authors:** José Manuel Trinidad, Ana Isabel Carnota, Inmaculada Failde, Luis Miguel Torres

**Affiliations:** ^1^Pain Unit, Department of Anesthesiology and Critical Care, University Hospital “Puerta del Mar”, Avenida Ana de Viya 21, 11009 Cádiz, Spain; ^2^Preventive Medicine and Health Public Area, University of Cádiz, Avenida Ana de Viya 52, 11009 Cádiz, Spain; ^3^Surgery Department, University of Cádiz, Plaza Fragela 9, 1003 Cádiz, Spain

## Abstract

*Study Design*. Quasiexperimental study.* Objective*. To investigate whether radiofrequency treatment can preclude the need for spinal surgery in both the short term and long term.* Background*. Radiofrequency is commonly used to treat lumbosacral radicular pain. Only few studies have evaluated its effects on surgical indications.* Methods*. We conducted a quasiexperimental study of 43 patients who had been scheduled for spinal surgery. Radiofrequency was indicated for 25 patients. The primary endpoint was the decision of the patient to reject spinal surgery 1 month and 1 year after treatment (pulsed radiofrequency of dorsal root ganglion, 76%; conventional radiofrequency of the medial branch, 12%; combined technique, 12%). The primary endpoint was the decision of the patient to reject spinal surgery 1 month and 1 year after treatment. In addition, we also evaluated adverse effects, ODI, NRS.* Results*. We observed after treatment with radiofrequency 80% of patients rejected spinal surgery in the short term and 76% in the long term. We conclude that radiofrequency is a useful treatment strategy that can achieve very similar outcomes to spinal surgery. Patients also reported a very high level of satisfaction (84% satisfied/very satisfied). We also found that optimization of the electrical parameters of the radiofrequency improved the outcome of this technique.

## 1. Background

Lumbar-radicular pain is a condition commonly encountered in pain units, and it represents one of the main reasons why patients request a consultation [1]. Conservative treatment (pharmacotherapy or physiotherapy) is effective in as many as 60% of patients, yet pain becomes chronic in the remaining cases and it produces a high degree of disability and mounting health care costs [1, 2]. Spinal surgery is the treatment of choice in these cases but this procedure is associated with recurring pain (postlaminectomy syndrome) in 10–40% of cases [3]. Accordingly, other minimally invasive techniques have been developed in recent years associated with a lower rate of associated complications [4].

Radiofrequency is the most widely used one of these procedures, due to its low complication rate (<1%), its ease of application, and the low associated costs [5]. The two most commonly used methods are conventional or thermal radiofrequency (CRF), in which a lesion is induced by heat generated by the vibration of particles and pulsed radiofrequency (PRF), whereby an electromagnetic field generated in the needle tip induces a series of changes at the cellular level that hinder action potential transmission by neurons [6].

Numerous studies have demonstrated the short- and long-term effectiveness of both procedures. However, a common concern of patients considering radiofrequency is whether this treatment will allow them to avoid surgery. A review of the literature reveals only one study that analyzed the impact of these procedures on surgery (a retrospective study on only 12 patients: [7]). Thus, our main goal in this study was to determine the percentage of patients, already scheduled for spinal surgery, that could avoid undergoing surgery by receiving lumbar radiofrequency (CRF of the medial branch and PRF of the dorsal root ganglion). In addition, we analyzed the effects of radiofrequency on pain intensity, the functional capacity of the patient, and their analgesic drug consumption, and we evaluated patient satisfaction and the side effects associated with this procedure.

## 2. Materials and Methods

### 2.1. Study Design

We used a quasiexperimental before-and-after study design. The participants were all patients that had been scheduled for spinal surgery by the Department of Neurosurgery at the University Hospital “Puerta del Mar” as of May 1, 2011 (*n* = 43), and who had previously undergone conservative pharmacological treatment and physiotherapy without success. Lumbar fusion had been indicated for 40% of the patients and laminectomy with discectomy for the remaining 60%. The study team was not involved at any stage in determining the surgical indication for these patients. The following exclusion criteria were applied: clinical/radiological discordance, large hernia, extrusion-migration and stenosis with bilateral claudication. The radiofrequency procedure was fully explained to all participating patients and their signed informed consent was obtained in all cases.

### 2.2. Study Protocol

The study was carried out over 4 consultations: Visit 1, baseline screening; Visit 2, radiofrequency treatment; Visit 3 (1-month posttreatment evaluation); and Visit 4 (1-year posttreatment evaluation). A total of 42 patients (1 failed to attend) underwent a clinical examination and an imaging test during the initial screening visit, which resulted in the exclusion of 13 patients based on the exclusion criteria defined in the study. In addition, three patients refused to undergo the procedure. Thus, the final number of patients included in the study was 26 (61.9%), with a mean age of 51 years (SD, 15.7) and 57.7% of them being male.

The following variables were evaluated during the visits before treatment (1) and those 1 month (3) and 1 year (4) after undergoing the procedure.

- NRS (Numeric Rating Scale) is a rating scale of pain intensity in which the patients rate their pain on a scale of 0 (no pain) to 10 “worst pain imaginable.” Accordingly, pain intensity was classified as mild (1–4), moderate (5-6), or severe (7–10).
- Oswestry Disability Index (ODI) is a questionnaire designed to determine the degree to which pain interferes with the performance of daily activities. The questionnaire consists of 10 items that are rated on a scale of 0–5 (minimum to maximum impairment). On completing the test, the points are added, divided by 50, and multiplied by 100 to obtain the percentage disability. The higher the ODI score, the greater the interference of pain.

The following variables were also evaluated at Visit 4:

- patient satisfaction scale: as measured using a 4-point verbal rating scale where 0 = “very dissatisfied,” 1 = “dissatisfied,” 2 = “neutral,” 3 = “satisfied,” and 4 = “very satisfied,”
- analgesic drug consumption: in which the patient's intake of analgesic medication was measured at the baseline, before treatment, and again at Visit 4 to determine the effect of treatment on analgesic drug consumption,
- adverse effects diary.

On Visits 3 and 4, the patients were asked to decide, depending on their clinical improvement, whether they would remain on the waiting list for surgery or reject undergoing surgery on their spine. In addition, on Visit 4 the number of patients who refused surgery on Visit 3 but ended up undergoing spinal surgery in the Neurosurgery Department between Visits 3 and 4 was recorded. The resulting data allowed us to determine how many of the patients treated with radiofrequency avoided spinal surgery in the short term (1 month) and long term (1 year), which is the main variable.

The breakdown of the spinal injuries suffered by the patients was L3-L4, 1 patient; L4-L5, 8 patients; L5-S1, 10 patients. Two patients presented a combined lesion at L4-L5 and L5-S1, while in one 3-facet involvement was observed and in another, canal stenosis with bilateral involvement of L5 due to listhesis of L4 over L5 was also described (Table 1).

### 2.3. Description of the Procedure

**Figure 1:**
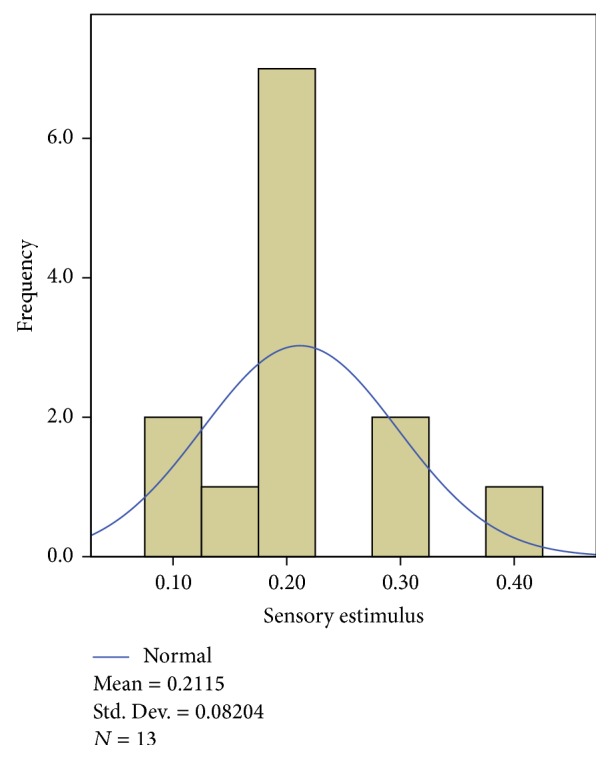
Histogram showing sensory stimulus values obtained for pulsed radiofrequency of the dorsal ganglion.

**Figure 2:**
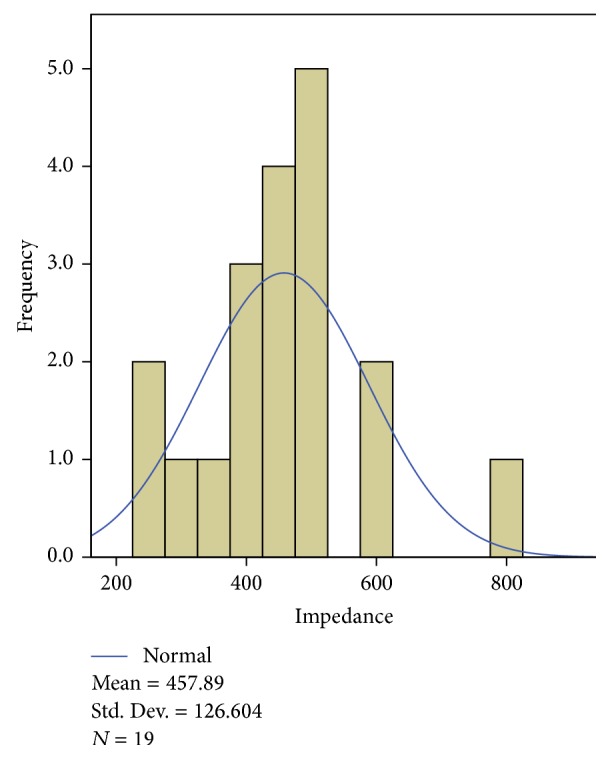
Histogram showing the impedance values obtained.

The patients that participated in this study were treated with PRF of the dorsal root ganglion (76%), CRF of lumbar medial branch (12%), or a combination of both techniques (12%). All procedures were performed under fluoroscopic guidance following radiation safety standards. The treatment procedures involving the dorsal root ganglion targeted the following roots: S1 (*n* = 13), L5 (*n* = 11), and/or L4 (*n* = 8). As reflected, in some cases, treatment targeted two roots that were causing pain. The optimal location was determined using contrast radiography and with electrical sensory (50 Hz) and motor (2 Hz) stimulation. Strict localization criteria were used, with an average sensory stimulation of 0.21 V (SD, 0.082) and an average impedance of 457.89 ohms (SD, 126.60) (Figures 1 and 2). A 100 mm SMK needle with a 5 mm active tip was used and a pulsed lesion was generated by applying 45 V for 6 minutes. The temperature reached never exceeded 42°C.

Facet pain was treated by conventional radiofrequency of the L3–L5 medial branch blocks. The procedure was carried out using radio guidance and positive neurostimulation, and it was directed at the junction of the superior articular pillar with the transverse process. A 100 mm SMK needle with a 10 mm active tip was used, and a conventional lesion was induced for 120 seconds at 25 V, reaching a temperature 70–80°C.

A URF-3AP radiofrequency generator (OWL upper range) with temperature control was used in all procedures.

### 2.4. Statistical Analyses

We performed a descriptive analysis of the data, calculating frequencies or measures of central tendency and the dispersion in function of the type of variable analyzed. A paired Student's *t*-test was used to compare pain intensity before and after the intervention and to analyze the ODI scores.

## 3. Results

Of the 42 patients scheduled for spinal surgery, 26 (61.9%) were selected for inclusion in the study in the screening visit. One of these patients could not tolerate the prone position required to perform the procedure and thus, this patient was dropped out of the study. The mean age of the 25 patients selected was 50.64 years (SD 15.92) and 56% were male. The mean baseline Visual Analogue Scale (VAS) was 7.64 (SD, 1.25) and the mean ODI score was 51.08% (SD, 14.43).

### 3.1. Rejection of Spinal Surgery

**Figure 3:**
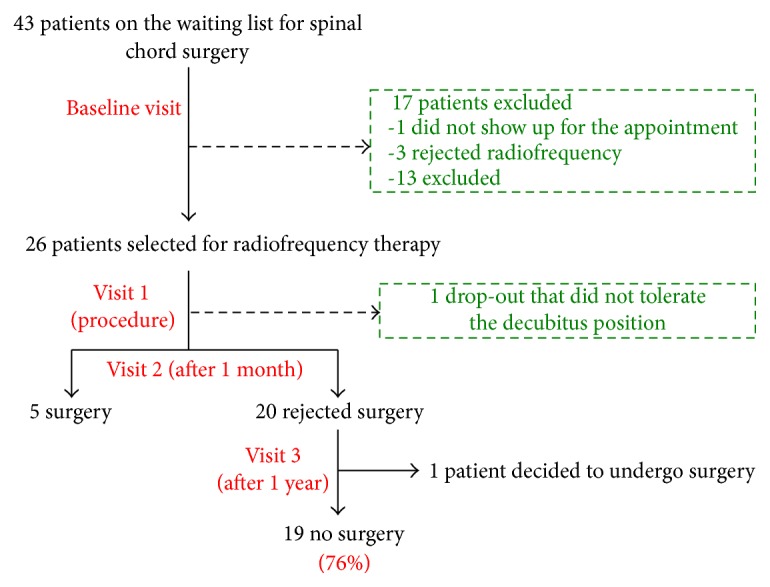
Flow chart outlining the study protocol.

At Visit 1 (1 month after treatment), 20 (80%) of the 25 patients studied refused to undergo the spinal surgery scheduled, representing a total decrease of 46.51% in the number of patients requiring spinal surgery initially. In the evaluation performed 1 year after radiofrequency, we found that only one of the patients treated who had opted out of surgery at the 1-month evaluation subsequently required a surgical intervention. This patient reported recurring back pain but no recurrence of radicular pain. Thus, 1 year after radiofrequency treatment, 19 of the 25 patients did not require surgery and as such, 76% of the patients treated with the minimally invasive technique (radiofrequency) had a favorable long-term outcome and avoided surgery (Figure 3). Indeed, our follow-up after radiofrequency treatment at the time of writing has reached 18 months, and the results are in line with those observed after a 1-year evaluation.

### 3.2. NRS (Numeric Rating Scale)

**Figure 4:**
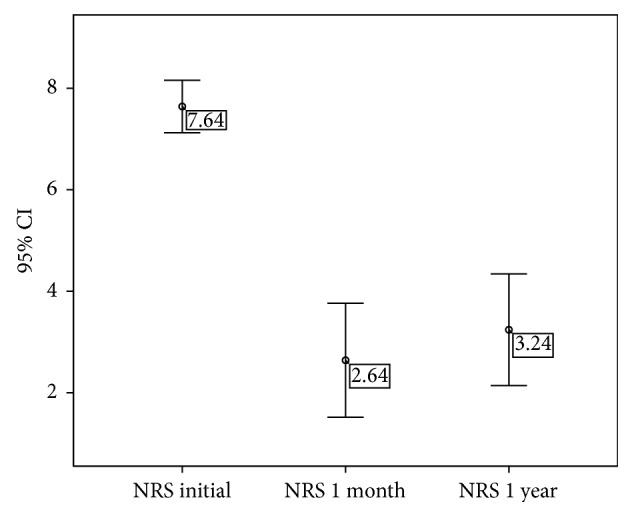
Evolution of the mean Numeric Rate Scale (NRS) score. Values represent the mean value ±95% CI.

**Figure 5:**
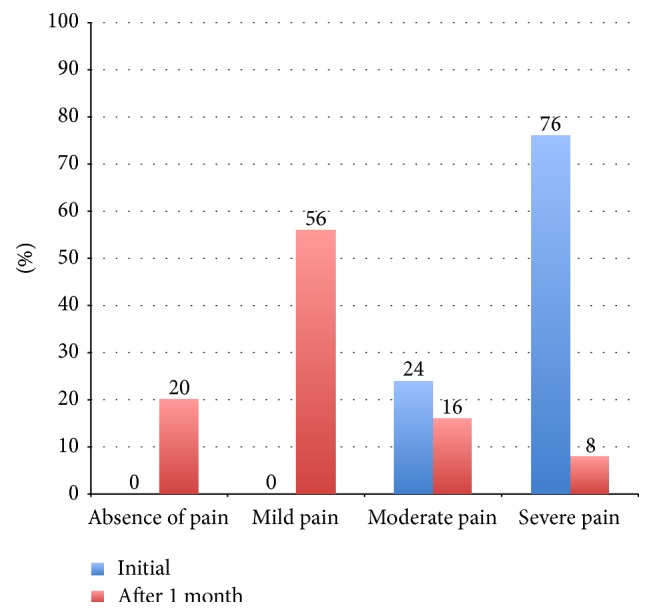
Classification of patients according to NRS score (%).

Initially, the patients selected presented a mean NRS score of 7.64 (95% CI, 7.12–8.16), whereas one month after radiofrequency treatment we observed a significant decrease (*p* < 0.01) in pain intensity, with an average NRS score of 2.64 (95% CI, 1.52–3.76). This difference persisted 1 year after treatment (mean NRS = 3.24; 95% CI, 2.14–4.34) (Figure 4). When the pain intensity in each group was analyzed, a decrease in the number of patients reporting “severe pain” decreased from 76% at baseline to 8% 1 month after treatment (Figure 5).

### 3.3. Oswestry Disability Index (ODI)

**Figure 6:**
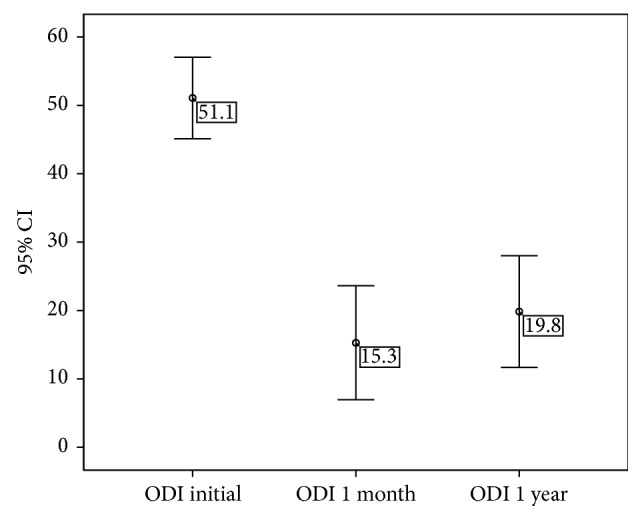
Evolution of Oswestry Disability Index (ODI) score. Values represent the mean value ±95% CI.

The 25 patients selected for the study had a basal ODI score of 51.08% (95% CI, 45.12–57.04), reflecting moderate impairment. One month after radiofrequency treatment (Visit 3), the mean ODI score for this same group was 15.28% (95% CI, 6.94–23.62), which differed significantly from the pretreatment score (*p* < 0.01). Moreover, this difference relative to the pretreatment score was maintained at 1 year after treatment (mean ODI score = 19.84%; 95% CI, 11.68–28.00; Figure 6).

### 3.4. Patient Satisfaction Scale

When patient satisfaction was measured at Visit 4 using a 5-point verbal rating scale, 36% of patients were satisfied and 48% were very satisfied. Thus, 84% of patients who underwent radiofrequency achieved a significant level of satisfaction (Table 2).

### 3.5. Decrease in Analgesic Drug Consumption

At the baseline, all the patients were being treated with analgesic drugs (NSAIDs, opioids, and neuromodulators), yet by Visit 4 (1 year after treatment), 68% of patients had decreased their consumption of analgesics with respect to the baseline levels and almost 35% achieved a complete cessation of medication.

### 3.6. Adverse Effects

No adverse effects were observed after radiofrequency treatment, although a few patients reported mild pain at the puncture site in the days following the treatment. This discomfort was resolved spontaneously without any need for further treatment.

## 4. Discussion

Lower back pain (with or without radiculopathy) is increasingly prevalent and indeed, it is the most common noncancer related pain pathology reported in pain units and in many cases, it is chronic in nature [1, 8]. Surgical treatment of this condition (laminectomy, microdiscectomy, and spinal fusion) has a success rate of between 60 and 80%. Nevertheless, the most feared complication is postlaminectomy syndrome or failed back surgery syndrome, the incidence of which ranges from 10 to 40% [3]. These statistics highlight the need for alternative treatments that have a similar success rates but with lower rates of associated complications [8].

Currently, two electrical procedures are commonly used to treat radicular and/or lower back pain: conventional radiofrequency of the medial branch for facet pain and pulsed radiofrequency of the dorsal root ganglion for radicular syndrome [9, 10].

Many studies have demonstrated the analgesic efficacy of these procedures. Van Kleef et al. reported the use of CRF has been attributed a level of evidence of 1B to treat lumbar facet syndrome [11], while pain relief of over 50% was reported by Van Boxem et al. in 13.1% of the patients after 1 year in a study of the use of PRF [4]. The effectiveness of this intervention in the short term has also been demonstrated in two prospective studies [8, 12]. However, other studies found no significant improvements with PRF-DRG [13]. Nevertheless, few studies have examined the direct impact of these procedures on surgical indications and indeed, the only such study is a retrospective study in which 12 patients scheduled for spinal surgery were followed up for 11 months [7]. However, in all but one of these patients good results were reported following radiofrequency treatment.

In our research, we found that 80% and 76% of patients who underwent radiofrequency treatment decided to refuse scheduled spinal surgery 1 month and 1 year after treatment, respectively. Moreover, treated patients reported a higher level of satisfaction (84% satisfied or very satisfied) and a decrease in the consumption of analgesics. Also, the low complication rate and a low cost should be noted. Together with the observed decrease in the number of surgical procedures performed in patients with lumbar radicular pain resistant to other treatments, these findings suggest that radiofrequency could be a useful alternative to surgery in certain circumstances.

It should be emphasized that the pathophysiology of radicular pain due to hernia involves* a mechanical and an inflammatory component*. The former is generated by direct compression of the nerve root by the hernia, while in the case of the latter, proinflammatory products of both the nucleus pulposus of the intervertebral disc and impaired venous and lymphatic drainage promote inflammation of the nerve root. Various studies have shown that pulsed radiofrequency can induce alterations in membrane and intracellular structures, thereby modifying action potential transmission and the perception of pain [14]. However, poor outcomes are likely when compression of the root is the main causative factor; in these cases, surgery would be clearly indicated. Thus, in our study, we excluded all cases in which the mechanical component predominated (e.g., extrusion-migration of the hernia, large foraminal hernia, and severe canal stenosis), and we obtained very satisfactory results that overcame the need for surgery in a large proportion of cases.

Another factor that appears to have been important in our study was the precise location of the needle relative to the dorsal root ganglion; thereby the mean sensory stimulation obtained was 0.21 V (DT = 0.082), ruling out the presence for motor stimulation twice the sensory stimulus achieved. The sensory stimulus that produces a positive response is inversely proportional to the distance from the tip of the needle. Thus, if we maintain a positive stimulus at a low voltage, we can bring the tip of the needle closer to the dorsal ganglion and achieve a more effective electromagnetic field for pulsed radiofrequency [6].

The impact of sensory stimulation has been recently analyzed, reporting no significant difference between the values of sensory stimulation and the effectiveness of the technique [15]. However, only CRF of facets was studied and not PRF of the dorsal ganglion. Based on our findings, we believe that a positive sensory stimulus of less than 0.25 V could contribute to the good response observed after PRF of the dorsal root ganglion.

Some limitations of our study should be borne in mind, such as the small sample size and the lack of a control group. The latter was not possible as the patients included were already scheduled for surgery.

In summary, radiofrequency using precise parameters for electrical localization and applying specific inclusion criteria could produce very satisfactory outcomes in patients over both the short term and long term, in some cases avoiding the need for surgery. Indeed, we think it is necessary to take radiofrequency into account before scheduling a surgery. Thus, there are indications for radiofrequency as well as for surgery.

However, further controlled studies with larger sample sizes will be necessary to better determine the efficacy of these treatments [16, 17].

## Figures and Tables

**Table 1:** Diagnosis of patients selected for radiofrequency (*n* = 25).

	Frequency	Percentage
Herniated disc L3-L4	1	4
Herniated disc L4-L5	8	32
Herniated disc L5-S1	10	40
Herniated disc L4-L5 and L5-S1	2	8
Canal stenosis	1	4
Facet joint hypertrophy	3	12

**Table 2:** Patient satisfaction with radiofrequency treatment.

Patient satisfaction scale	Frequency	Percentage
Very dissatisfied	0	0
Dissatisfied	3	12
Neutral	1	4
Satisfied	9	36
Very satisfied	12	48

## References

[1] Konstantinou K., Dunn K. M. (2008). Sciatica: review of epidemiological studies and prevalence estimates. *Spine*.

[2] Weber H. (1993). The natural course of disc herniation. *Acta Orthopaedica Scandinavica*.

[3] Bosscher H. A., Heavner J. E. (2010). Incidence and severity of epidural fibrosis after back surgery: an endoscopic study. *Pain Practice*.

[4] Van Boxem K., van Bilsen J., de Meij N. (2011). Pulsed radiofrequency treatment adjacent to the lumbar dorsal root ganglion for the management of lumbosacral radicular syndrome: a clinical audit. *Pain Medicine*.

[5] van Boxem K., Cheng J., Patijn J. (2010). 11. Lumbosacral radicular pain. *Pain Practice*.

[6] Cosman E. R., Cosman E. R. (2005). Electric and thermal field effects in tissue around radiofrequency electrodes. *Pain Medicine*.

[7] Teixeira A., Grandinson M., Sluijter M. E. (2005). Pulsed radiofrequency for radicular pain due to a herniated intervertebral disc—an initial report. *Pain Practice*.

[8] Chao S.-C., Lee H.-T., Kao T.-H. (2008). Percutaneous pulsed radiofrequency in the treatment of cervical and lumbar radicular pain. *Surgical Neurology*.

[9] Chua N. H. L., Vissers K. C., Sluijter M. E. (2010). Pulsed radiofrequency treatment in interventional pain management: mechanisms and potential indications—a review. *Acta Neurochirurgica*.

[10] Nagda J. V., Davis C. W., Bajwa Z. H., Simopoulos T. T. (2011). Retrospective review of the efficacy and safety of repeated pulsed and continuous radiofrequency lesioning of the dorsal root ganglion/segmental nerve for lumbar radicular pain. *Pain Physician*.

[11] Van Kleef M., Vanelderen P., Cohen S. P., Lataster A., Van Zundert J., Mekhail N. (2010). 12. Pain originating from the lumbar facet joints. *Pain Practice*.

[12] Abejon D., Garcia-del-Valle S., Fuentes M. L. (2007). Pulsed radiofrequency in lumbar radicular pain: clinical effects in various etiological groups. *Pain Practice*.

[13] Shanthanna H., Chan P., McChesney J., Paul J., Thabane L. (2014). Pulsed radiofrequency treatment of the lumbar dorsal root ganglion in patients with chronic lumbar radicular pain: a randomized, placebo-controlled pilot study. *Journal of Pain Research*.

[14] Erdine S., Bilir A., Cosman E. R., Cosman E. R. (2009). Ultrastructural changes in axons following exposure to pulsed radiofrequency fields. *Pain Practice*.

[15] Cohen S. P., Strassels S. A., Kurihara C. (2011). Does sensory stimulation threshold affect lumbar facet radiofrequency denervation outcomes? A prospective clinical correlational study. *Anesthesia & Analgesia*.

[16] Gallagher R. M. (2005). Pulsed radiofrequency treatment: biological mechanisms and clinical evidence. *Pain Medicine*.

[17] Van Boxem K., Joosten E. A., van Kleef M., Patijn J., van Zundert J. (2012). Pulsed radiofrequency treatment for radicular pain: where do we stand and where to go?. *Pain Medicine*.

